# Rivaroxaban Counteracts the Adverse Effects of Warfarin on Plaque Vulnerability and Vessel Wall Remodelling

**DOI:** 10.1055/a-2979-1901

**Published:** 2026-10-21

**Authors:** Anxhela Habibi, Vanessa Broker, Juliana H. Boukhaled, Dani Veldhuis, Jenny Surholt, Peter Leenders, Cecile Maassen, Magdolna Nagy, Henri M.H. Spronk, Wolfram Ruf, Leon J. Schurgers, Armand M.G. Jaminon

**Affiliations:** 1 Department of Biochemistry 5211 Cardiovascular Research Institute Maastricht, Maastricht UniversityMaastrichtLimburgThe Netherlands; 2 39068 Center for Thrombosis and Hemostasis (CTH), University Medical Center of the Johannes Gutenberg University MainzMainzRhineland-PalatinateGermany; 3 Department of Nephrology 27037 University of MontpellierMontpellierOccitanieFrance; 4 199236 Thrombosis Expertise Center, Heart and Vascular Center, Maastricht University Medical Centre+MaastrichtLimburgThe Netherlands

**Keywords:** atherosclerosis, vascular calcification, warfarin, rivaroxaban, plaque stability, oral anticoagulants

## Abstract

Atherosclerosis is a major cause of cardiovascular diseases (CVDs) globally, characterized by vascular calcification (VC), increasing the risk of myocardial infarction and stroke. Oral anticoagulants are widely prescribed to prevent recurrent thrombotic events. However, increasing concern surrounds warfarin, whose unfavorable pharmacokinetic profile and interference with vitamin K-dependent pathways have been implicated in VC. Rivaroxaban represents an alternative anticoagulant, yet its ability to mitigate warfarin-induced vascular injury remains insufficiently defined. This study investigated whether switching from warfarin to rivaroxaban benefits plaque progression and might hold progression of calcification. Apolipoprotein E-deficient ( *Apoe ^−/−^* ) mice were fed a Western-type diet (WTD) for 18 weeks, supplemented without (control) or with either warfarin or rivaroxaban. An additional group received warfarin for 6 weeks, after which they switched to rivaroxaban for 12 weeks. Plaque size was comparable in both the continuous rivaroxaban group and the switching group and was decreased compared with 18 weeks in both the warfarin and control groups. Notably, VC was decreased in both rivaroxaban groups, with a more significant decrease observed in the switching group compared with continuous warfarin treatment. The switching group showed better vascular integrity with lower vascular stiffness and fewer elastic fiber breaks. Additionally, the switching group showed features of a more stable plaque phenotype with increased α-smooth muscle actin (αSMA)-positive vascular smooth muscle cells and a higher collagen content. Interestingly, all three anticoagulation therapies showed decreased expression of protease-activated receptor 2 (PAR2) together with a decrease in oxidative stress in the plaques. However, only rivaroxaban treatments showed reduced matrix metalloproteinase 9 (MMP-9) expression in plaques compared with both control and warfarin groups. Our results suggest that rivaroxaban ameliorates the adverse effects of warfarin, offering significant benefits on atherosclerotic burden and plaque stability. Transitioning from warfarin to rivaroxaban has the potential to reshape clinical practice by offering anticoagulation and reducing the practical limitations associated with vitamin K antagonists, particularly in patients with coexisting CVDs.

## Introduction

According to the World Health Organization (WHO), cardiovascular diseases (CVDs) are the leading cause of mortality worldwide, with atherosclerosis being the underlying cause of most CVDs. 1 Atherosclerosis is a chronic inflammatory disease characterized by the accumulation of lipoproteins and inflammatory cells within the intima of the arterial vessel wall and the migration of vascular smooth muscle cells (VSMCs) from the media into the intima, resulting in an atherosclerotic plaque. This process leads to luminal narrowing and vessel wall stiffening, resulting in reduced blood flow and increased risk for cardiovascular events, such as stroke or myocardial infarction. 2

The composition of the atherosclerotic plaque affects its stability. Vulnerable plaques can rupture and cause atherothrombosis, responsible for the majority of acute coronary syndrome (ACS). 3 Vascular calcification (VC), in particular microcalcifications (<50 μm), confer a more unstable plaque phenotype by accumulating mechanical stress that can lead to plaque rupture. 4 Alongside VC, oxidative stress and endoplasmic reticulum stress orchestrate macrophage apoptosis and defective efferocytosis, leading to necrotic core expansion and increased plaque vulnerability. 5 Matrix metalloproteinase 9 (MMP-9) degrades key components of the extracellular matrix (ECM), such as collagen IV and elastin, and has been consistently identified as a clinical predictor of atherosclerotic plaque instability. 6 By mediating elastolysis and collagenolysis, MMPs further compromise fibrous cap integrity, thereby amplifying the risk of plaque rupture. 7

Warfarin has long been used as an anticoagulant but is associated with several limitations, including variable pharmacokinetics, numerous drug and dietary interactions, and the need for regular monitoring due to its narrow therapeutic window. In addition, concerns have been raised regarding warfarin’s potential role in promoting VC and increasing atherosclerotic plaque burden. 8 These challenges led to the development of direct oral anticoagulants (DOACs). Rivaroxaban, a direct Factor Xa (FXa) inhibitor, is used to prevent venous thromboembolism in patients undergoing planned hip or knee replacement surgery, treat deep vein thrombosis and pulmonary embolism, reduce the risk of stroke in patients with non-valvular atrial fibrillation, and prevent atherothrombotic events following ACS. 9 The effect of rivaroxaban on atherosclerosis has been studied in animal models where rivaroxaban treatment reduced atherosclerotic lesions and promoted regression of existing atherosclerotic plaques in apolipoprotein E-deficient ( *Apoe ^−/−^* ) mice fed a Western-type diet (WTD). 10 Inhibiting FXa also decreased the expression of its main receptor, protease-activated receptor 2 (PAR2). Additionally, mouse models deficient in PAR2 have shown that targeting PAR2 can ameliorate atherosclerosis by blunting the expression of chemokine-induced monocyte migration in plaques. 11

While Posthuma et al 10 showed that rivaroxaban stimulates plaque regression in mice fed WTD, little is known about the potential effect of switching from warfarin to rivaroxaban on the vasculature. Therefore, this study aims to compare the effects of warfarin and rivaroxaban on plaque formation, plaque composition, and vessel wall remodelling and to assess whether switching from warfarin to rivaroxaban counteracts the detrimental vascular effects of warfarin in an *Apoe ^−/−^* mouse model. Clinically, switching from warfarin to DOACs is currently being implemented due to their superior efficacy, more favorable adverse effect profile, fewer drug–drug interactions, and the absence of the need for international normalized ratio (INR) monitoring. 12 Understanding the distinct vascular effects of warfarin and rivaroxaban is critical for optimizing anticoagulation strategies in elderly patients, who often present with substantial cardiovascular comorbidity and are particularly vulnerable to treatment-related vascular complications. As clinical practice increasingly shifts from warfarin to DOACs, a clearer understanding of their divergent vascular effects is needed. Such insights will enable the development of therapeutic strategies, ultimately improving outcomes and guiding more effective management of complex CVDs.

## Methods

### Animals

Ten- to 12-week-old female C57BL/6J *Apoe ^−/−^* mice were purchased from Charles River and kept in climate-controlled facilities with a 12-hour light/dark cycle with ad libitum access to food and water. All experiments conducted were approved by the ethics committee for animal experiments of Maastricht University.

Animals received a WTD for 18 weeks (0.25% cholesterol; Sniff) either alone or supplemented with warfarin (3 mg/g [Merck KGaA] + 1.5 mg/g vitamin K1) or rivaroxaban (1.2 mg/g, Bayer AG). Additionally, a switching group was implemented in which animals received warfarin for the first 6 weeks, followed by rivaroxaban for 12 weeks. For the WTD, warfarin, and rivaroxaban groups, a subset of mice was sacrificed at 6 weeks, and the remaining mice, including the switching group, were sacrificed at 18 weeks ( Fig. 1A ). Blood was collected in 3.2% trisodium citrate tubes via the portal vein. Platelet-poor plasma aliquots were prepared by centrifugation (500× *g* for 15 min at room temperature) and frozen at −80 °C until analysis. Next, the vasculature was washed with 100 μM sodium nitroprusside in phosphate-buffered saline (PBS). The aortic arch, brachiocephalic artery, left common carotid artery, and left subclavian artery were excised, including surrounding adipose and connective tissues. The tissue was fixed in 1% paraformaldehyde overnight before paraffin embedding for subsequent analysis.

### Plasma Cholesterol Measurement

Total plasma cholesterol levels were assessed using the ABX Pentra Cholesterol CP reagent (HORIBA ABX). Absorbance (490 nm wavelength), which is proportional to the cholesterol concentration, was measured with the Cytation 3 Imaging system (BioTek) and compared with a standard cholesterol calibration curve to determine the sample concentration.

### Thrombin Generation

Thrombin generation measurement by means of the Calibrated Automated Thrombogram 13 (Stago BNL) was performed in 1:4 diluted plasma using PPP reagent (Stago BNL). Thrombin generation curves were recorded for 90 min at 37 °C following a 10-minute preincubation at 37 °C. Peak height and endogenous thrombin potential (ETP) were calculated by Thrombinoscope Software (Thrombinoscope BV).

### Pulse Wave Velocity

Pulse wave velocity (PWV) in the right common carotid artery and the abdominal aorta was measured using the transit-time method in a two-dimensional mode. The transit time was calculated as PWV = Δ *d* /( *Pt*_dist_ − *Pt*_prox_ ), where *Pt* is the time point of the proximal or distal pulse wave signal, and Δ *d* is the distance between the two measuring points. ΔPWV was measured as PWV ( *t* = 18 − *t* = 0 weeks).

### Histology

To analyze plaque characteristics, multiple histochemical stains were performed. The tissue was serially sectioned at 4-μm thickness to enable analysis of the three-dimensional structure. The paraffin block was cut to a suitable size, attached to the microtome, and the surface of the block was trimmed to expose the tissue. Cut sections were transferred to a water bath before being placed on adhesive microscope slides. After the whole tissue was cut, the slides were dried at 37 °C for 24 h.

Prior to all staining, tissue sections were deparaffinized and rehydrated through a graded alcohol series (two times xylol, two times 100% ethanol, two times 96% ethanol, 70% ethanol, 50% ethanol, and demineralized water), and antigen retrieval was performed. Plaque size in mouse aortic arches was determined using hematoxylin and eosin (H&E) staining. Plaque characteristics were quantified using Alizarin Red S (AR) for calcification, picrosirius red (PSR) for collagen content, and Elastica van Gieson (EvG) for elastic fibers. Collagen content was estimated using a scoring system ranging from 1 to 5 (1: 0–20%, 2: 20–40%, 3: 40–60%, 4: 60–80%, 5: 80–100%).

For immunohistochemical staining, slides were blocked and incubated overnight with primary antibodies as described: anti-PAR2 (1:1,000, kind gift from W.R.), anti-α-smooth muscle actin (αSMA, 1:500, 1A4 Dako, M0851), anti-MMP-9 (1:250, BioConnect, orb11064), and 8-hydroxyguanosine (1:300, Meridian, Q2B200G). Then, sections were incubated for 1 h with the appropriate HRP-conjugated secondary antibody and detected with NovaRED for 2–5 min (Vector Labs, SK-4800).

Slides were imaged using the Leica DM2000 microscope (Leica Microsystems GmbH) and the LAS X software (Leica Microsystems GmbH) or the Olympus BX61VS microscope (EVIDENT Europe GmbH) and the Olympus VS-ASW software (EVIDENT Europe GmbH). For quantification, the software ImageJ, QuPath 0.3.2, and MATLAB R2023b (MathWorks) were used.

The necrotic core size of atherosclerotic lesions was calculated using H&E-stained tissue sections and the software ImageJ. The total plaque area was measured using the unlimited threshold (UT), capturing the entire plaque area. Then, a specific threshold was applied to isolate the necrotic core. This limited threshold (LT) included only pixels within a defined intensity range.

A MATLAB script was used to quantify elastic fiber breaks in the aortic arch. The number of measured endpoints corresponded to the number of elastic fiber breaks within the selected region.

### Statistical Analysis

Data are presented as mean ± standard deviation (SD). Normality was assessed using the Shapiro–Wilk test. If datasets did not pass the normality test, Kruskal–Wallis with Dunn’s multiple comparisons test was performed. Multiple group comparisons were performed using one-way analysis of variance (ANOVA) with Tukey’s multiple comparison test. Statistical analyses were performed using GraphPad Prism version 10.2.2. (GraphPad). A two-sided *p* -value < 0.05 was considered statistically significant. Exact *p* -values are stated.

## Results

### Animal Model Validation

Animals were weighed at *t* = 6 weeks and *t* = 18 weeks ( Fig. 1B,D ). The warfarin group showed a decrease in weight compared with the other groups at *t* = 18 weeks, and a decreased weight compared with the WTD group at *t* = 6 weeks. No significant changes were observed in the rivaroxaban groups compared with the WTD group at both 6 and 18 weeks. Cholesterol levels were elevated in the warfarin group compared with the WTD group, with no differences noted in the rivaroxaban groups compared with the WTD group at *t* = 18 weeks and *t* = 6 weeks ( Fig. 1C,E ).

**Fig. 1:**
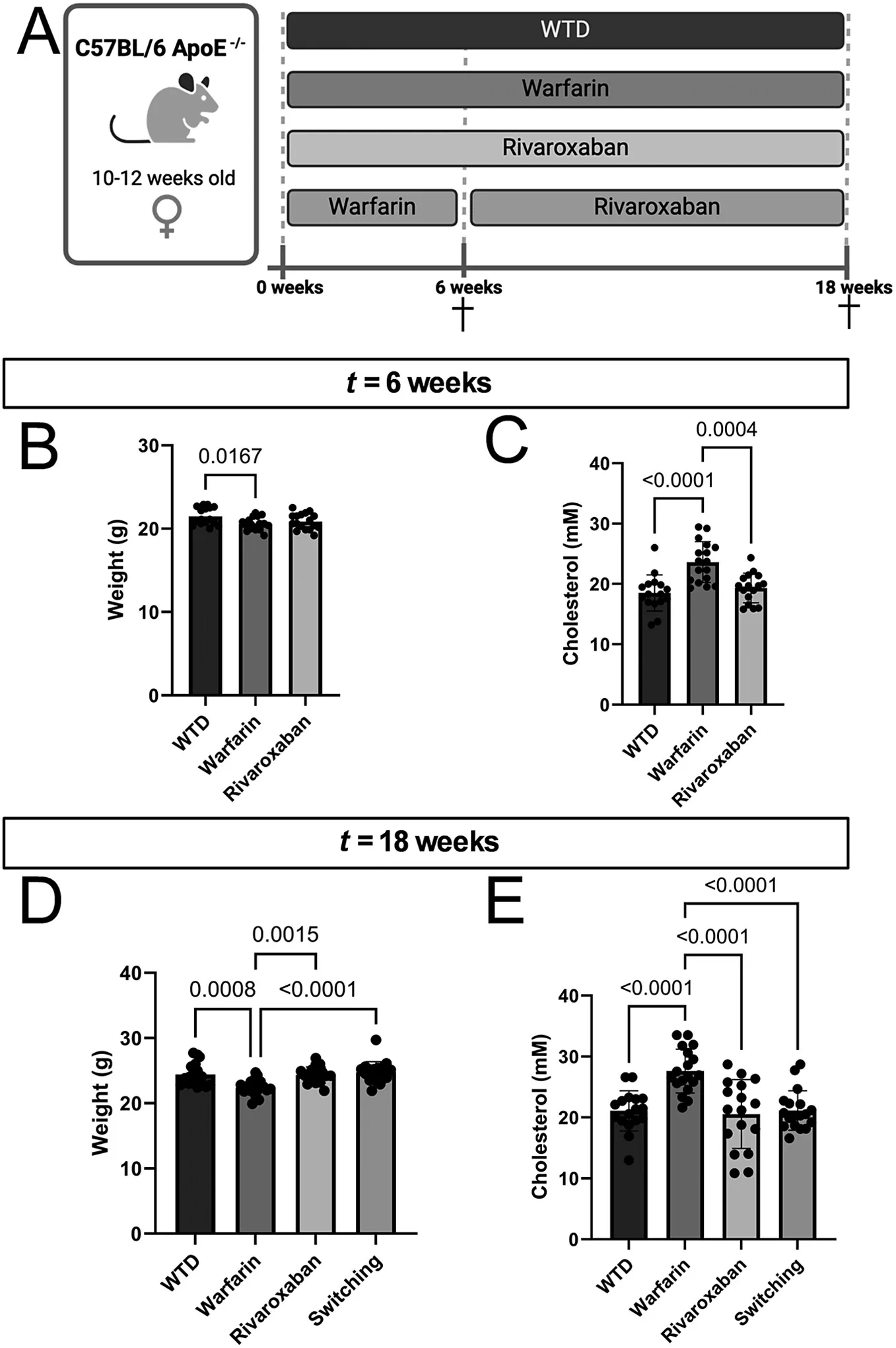
Study design and animal measurements. ( **A** ) Graphical representation of the animal groups and timeline. Sacrifice was performed after 6 weeks for WTD, warfarin, and rivaroxaban treatments and after 18 weeks for WTD, warfarin, and rivaroxaban and switching groups. ( **B** ) Weight (g) at *t* = 6 weeks for all groups. One-way ANOVA, Tukey’s multiple comparison test, *n* = 17. ( **C** ) Cholesterol measurements at *t* = 6 weeks. One-way ANOVA, Tukey’s multiple comparison test, *n* = 17–18. ( **D** ) Weight (g) at *t* = 18 weeks for all groups. One-way ANOVA, Tukey’s multiple comparison test, *n* = 17–18. ( **E** ) Cholesterol measurements at *t* = 18 weeks. One-way ANOVA, Tukey’s multiple comparison test, *n* = 17–18. [rerif]. Source: Created in BioRender. Schurgers, L. (2026) https://BioRender.com/naeb9jy . ANOVA, analysis of variance; *Apoe ^−/−^* , apolipoprotein E-deficient; WTD, Western-type diet.

Thrombin generation assay was performed to assess the anticoagulant effects of warfarin and rivaroxaban compared with the non-coagulation group (WTD). Endogenous thrombin potential (ETP) represents the area under the thrombin generation curve, thereby reflecting the total amount of thrombin generated over the analytical time. At *t* = 6 weeks, warfarin showed a numerical decrease in ETP compared with WTD and was significantly lower compared with rivaroxaban ( Fig. 2A ). At *t* = 18 weeks, ETP was reduced in the warfarin group compared with the WTD group, but no significant differences were observed for the rivaroxaban groups compared with WTD ( Fig. 2C ). The peak height indicates the maximum amount of thrombin generated. At *t* = 6 weeks, rivaroxaban treatment showed a significantly lower peak height compared with WTD and warfarin ( Fig. 2B ). At *t* = 18 weeks, the peak height was lower in the rivaroxaban and switching groups compared with both WTD and warfarin groups ( Fig. 2D ). Warfarin did not affect thrombin peak height relative to WTD at both *t* = 6 weeks and *t* = 18 weeks. The distinct curve morphologies observed ( Fig. 2E ) explain why warfarin, despite achieving a peak height comparable to WTD, resulted in a lower ETP. Specifically, the warfarin curve was narrower, reflecting a reduced overall thrombin generation. In contrast, the rivaroxaban groups displayed a prolonged onset time with lower peak heights than both warfarin and WTD; however, their curves were broader, resulting in no apparent change in ETP.

**Fig. 2:**
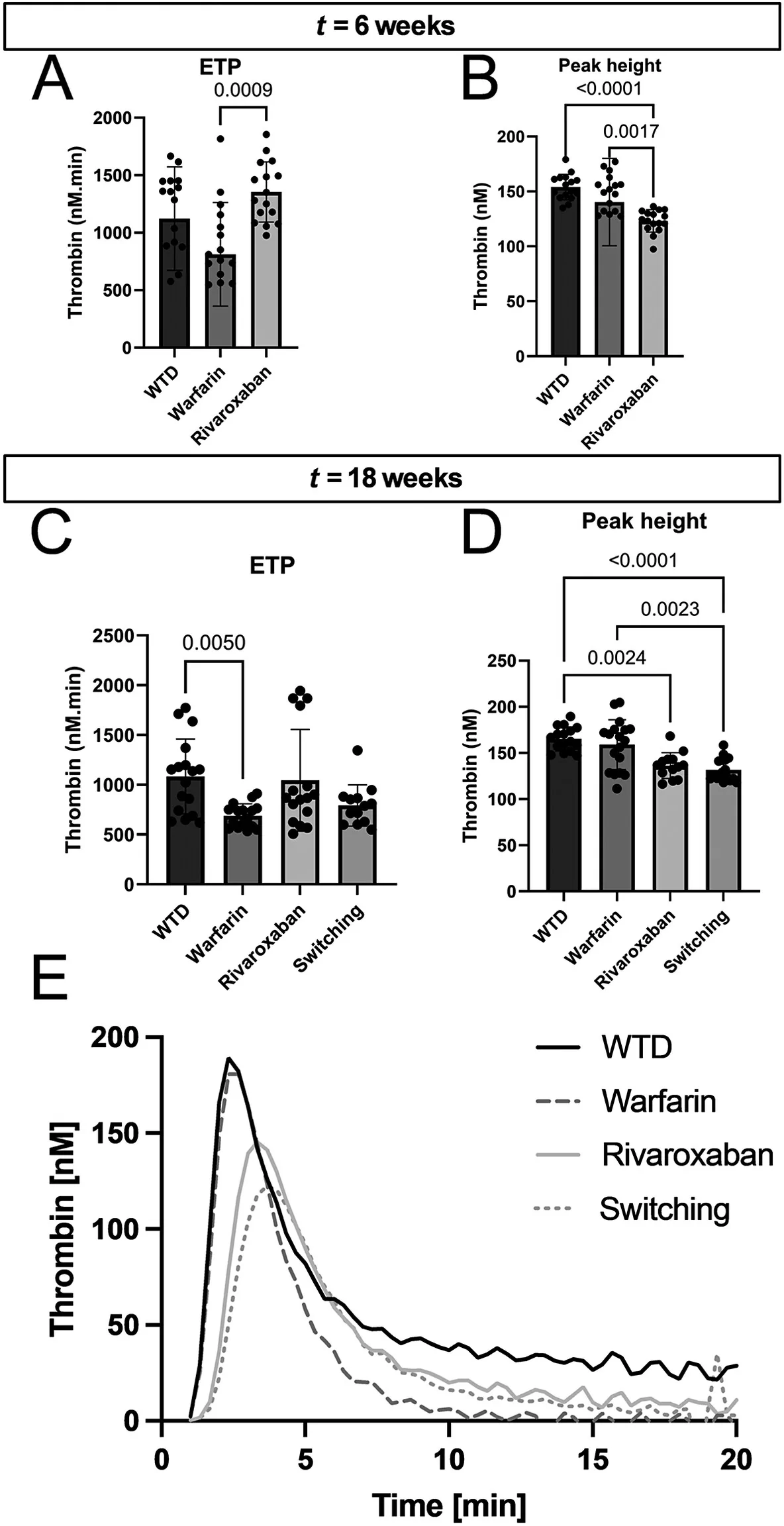
Anticoagulation efficacy at *t* = 6 weeks and *t* = 18 weeks. Thrombin generation was performed to assess anticoagulation therapy. Endogenous thrombin potential (ETP) is the overall thrombin generated over time, while peak height represents the highest concentration of thrombin generated. ( **A** ) ETP measured at *t* = 6 weeks. One-way ANOVA, Tukey’s multiple comparison test, *n* = 13–18. ( **B** ) Peak height measured at *t* = 6 weeks. Kruskal–Wallis with Dunn’s multiple comparison test, *n* = 13–18. ( **C** ) ETP measured at *t* = 18 weeks. Kruskal–Wallis with Dunn’s multiple comparison test, *n* = 13–18. ( **D** ) Peak height measured at *t* = 18 weeks. Kruskal–Wallis with Dunn’s multiple comparison test, *n* = 13–18. ( **E** ) Representative curves of thrombin generation at *t* = 18 weeks for all groups. ANOVA, analysis of variance; WTD, Western-type diet.

### Rivaroxaban Treatment Shows Decreased Plaque Size, Calcification, and Vascular Remodelling

To assess plaque size, aortic arches were stained with H&E ( Fig. 3A ). At 18 weeks, plaque size was significantly reduced in both rivaroxaban groups compared with the warfarin group. The switching group had significantly smaller plaque size compared with the WTD and warfarin groups ( Fig. 3B ). Interestingly, plaque size at 6 weeks was significantly greater in the warfarin-treated group compared with both the WTD and rivaroxaban groups ( Fig. 3C ).

**Fig. 3:**
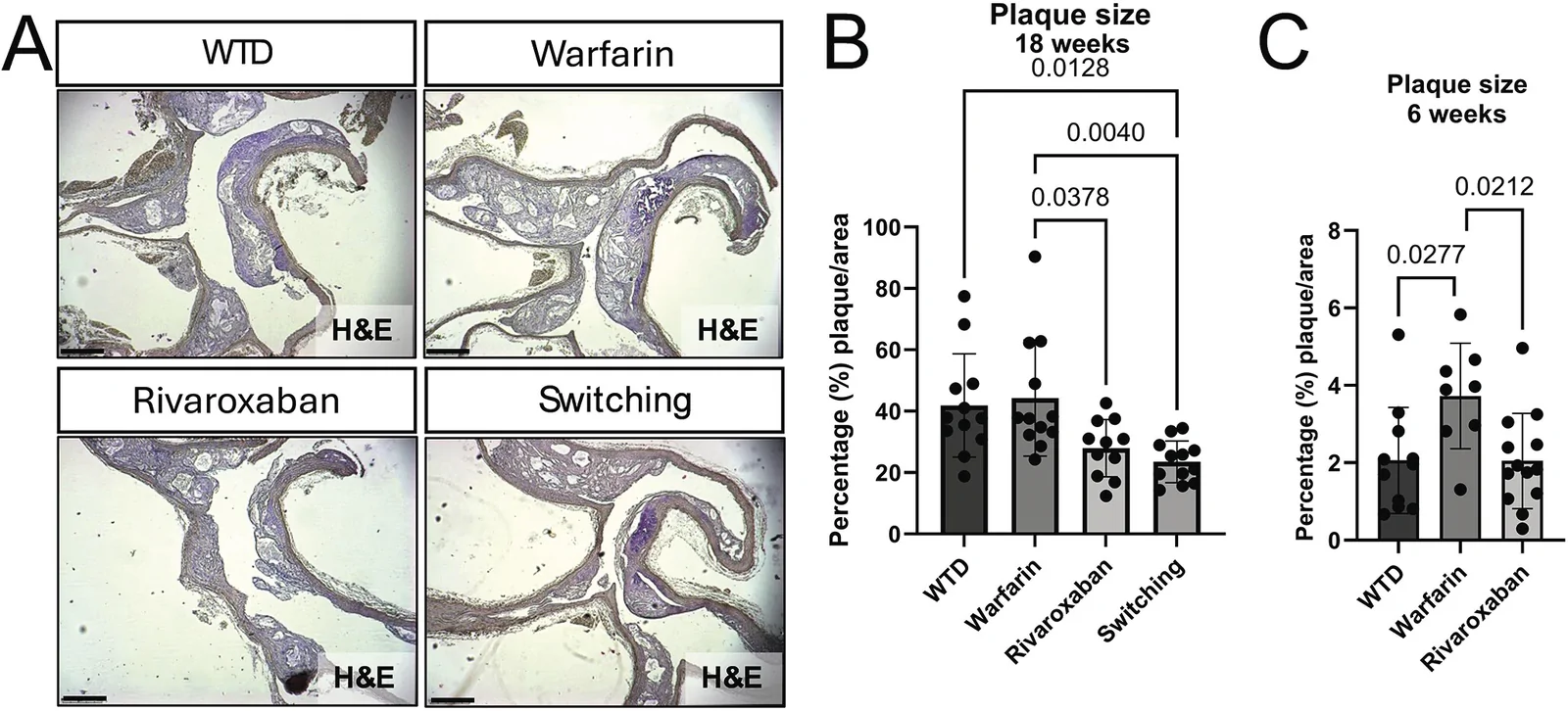
Plaque sizes at 6 and 18 weeks. ( **A** ) Representative pictures of hematoxylin and eosin (H&E) staining for all groups at 18 weeks. Scale bar = 250 μm. ( **B** ) Plaque size quantification at 18 weeks as a percentage of plaque over total tissue area. ( **C** ) Plaque size quantification of 6-week-treated animals. ( **B, C** ) One-way ANOVA, Tukey’s multiple comparison test, *n* = 11–12. ANOVA, analysis of variance; WTD, Western-type diet.

Additionally, aortic arches were stained with AR to visualize calcification ( Fig. 4A ). The warfarin group revealed increased calcification compared with the WTD group ( Fig. 4B ). The switching group showed significantly less calcification compared with the warfarin group. AR staining was negative in all groups treated for 6 weeks (data not shown).

**Fig. 4:**
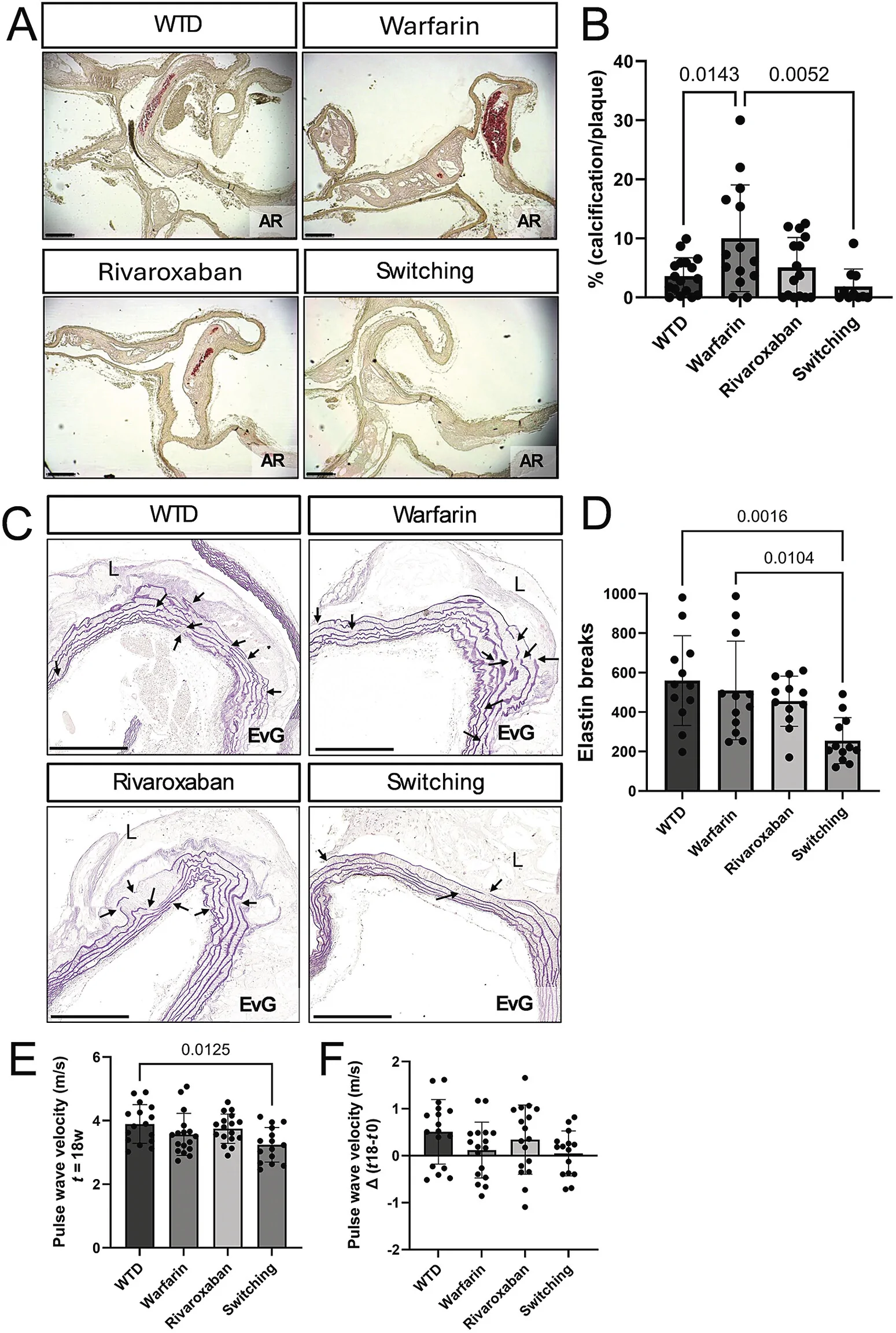
Vascular calcification and vessel wall remodelling. ( **A** ) Representative pictures of AR staining to detect calcification. Scale bar = 250 μm. ( **B** ) Quantification of AR staining is shown as a percentage of red signal per plaque. One-way ANOVA, Tukey’s multiple comparison test, *n* = 10–17. ( **C** ) Representative pictures of EvG staining of elastic fibers. Black arrows indicate breaks. Scale bar = 250μm. ( **D** ) Quantification of elastic fiber breaks under the aortic arch plaques as the number of endpoints. One-way ANOVA, Tukey’s multiple comparison test, *n* = 12. ( **E** ) PWV measurements to determine changes in vessel wall stiffness. One-way ANOVA, Tukey’s multiple comparison test, *n* = 15–17. ( **F** ) PWV calculated as Δ ( *t*_18_ − *t*_0_ ). One-way ANOVA, Tukey’s multiple comparison test, *n* = 15–17. ANOVA, analysis of variance; AR, Alizarin Red S; EvG, Elastica van Gieson; L, lumen; PWV, pulse wave velocity; w, weeks; WTD, Western-type diet.

To determine how the vessel wall is affected by the different anticoagulant treatments, aortic tissue was stained with EvG ( Fig. 4C ). The number of elastic breaks underlying the plaques was significantly reduced in the rivaroxaban switching group compared with the warfarin and the WTD groups ( Fig. 4D ).

PWV was measured to assess potential differences in vascular stiffness among the groups. At 18 weeks, only the switching group showed a significant decrease in PWV compared with the WTD group ( Fig. 4E ). Analysis of ΔPWV revealed group-dependent visual differences (although not reaching significance) in the temporal evolution of vascular stiffness indices, with the switching group showing a relative attenuation of PWV progression compared with WTD ( Fig. 4F ).

### Rivaroxaban Treatment Shows Increased Features of Plaque Stability

To explore the composition of atherosclerotic plaques under different anticoagulation therapies, aortic arches were stained for αSMA, a marker expressed by contractile VSMCs ( Fig. 5A ). Total αSMA in the aortic tissues showed a significant increase in the switching group compared with the WTD group ( Fig. 5B ). When αSMA was measured in the vascular wall, no significant differences were detected, despite a visual increase in the switching group compared with all other groups ( Fig. 5C ). However, αSMA in the plaques was significantly increased in the switching group compared with all other groups ( Fig. 5D ), with αSMA-positive VSMCs surrounding the plaque in the switching group ( Fig. 5A ).

**Fig. 5:**
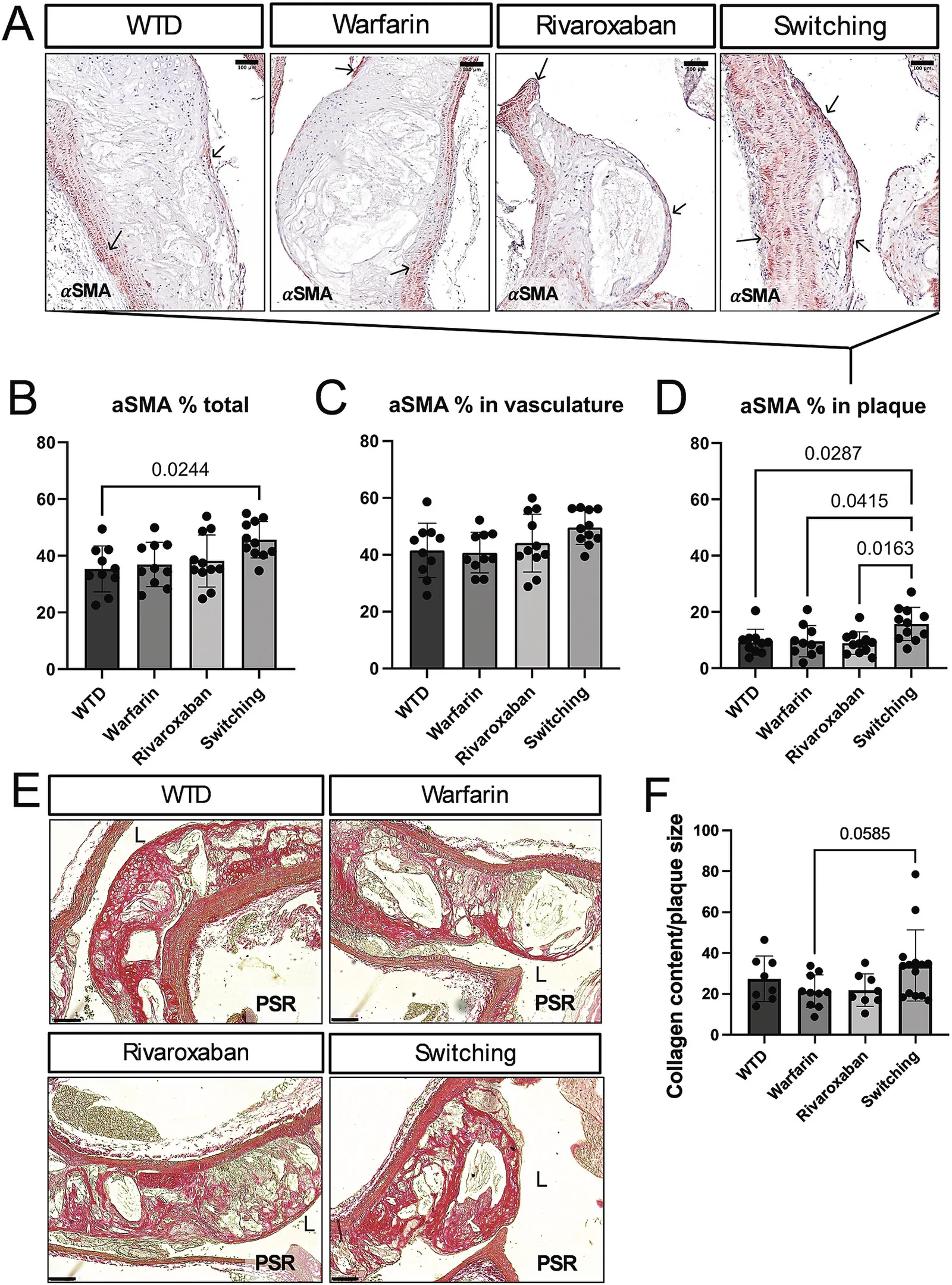
Plaque stability features: αSMA and collagen content. ( **A** ) Representative pictures of αSMA-stained mouse aortas, focusing on the plaque. Scale bar = 100 μm. Black arrows indicate examples of positive signal. ( **B** ) Quantification of αSMA as a percentage of signal over total tissue area. ( **C** ) Percentage of αSMA expressed only in the vasculature, calculated as total signal in the vessel wall. ( **D** ) αSMA expressed in the plaques as a percentage of signal over plaque size. One-way ANOVA, Tukey’s multiple comparison test, *n* = 10–11. ( **E** ) Representative pictures of PSR staining for collagen. Scale bar = 75 μm. ( **F** ) Quantification of collagen content normalized to plaque size. One-way ANOVA, Tukey’s multiple comparison test, *n* = 8–14. ANOVA, analysis of variance; PSR, picrosirius red; L, lumen; WTD, Western-type diet; αSMA, α-smooth muscle actin.

Since higher collagen content is considered to stabilize plaques, 14 aortic arches were stained with PSR to assess the collagen content in the plaques ( Fig. 5E ). In the switching group, the collagen content per plaque increased compared with the warfarin group, but this did not reach statistical significance ( Fig. 5F ).

Elevated MMP-9 expression in plaques has been associated with a more unstable plaque phenotype. 15 Staining for MMP-9 revealed that in both rivaroxaban groups, MMP-9 expression was significantly decreased ( Fig. 6A,B ).

**Fig. 6:**
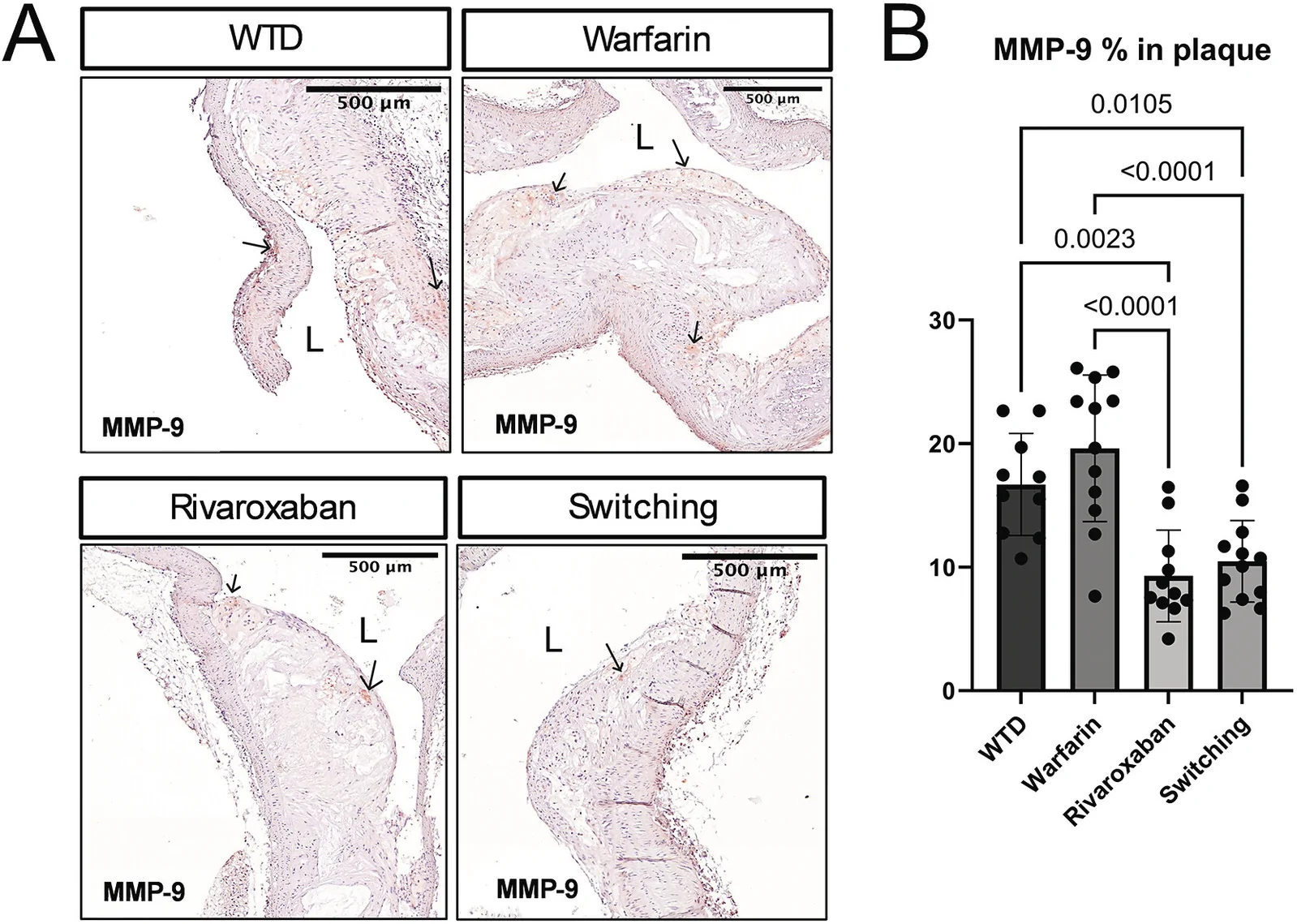
Plaque instability features: matrix metalloproteinase 9 (MMP-9) and oxidative stress. ( **A** ) Representative pictures of MMP-9-stained mouse aortic plaques. Scale bar = 500 μm. Black arrows indicate examples of positive signal. ( **B** ) Quantification of MMP-9 expression in the plaques as a percentage of signal over plaque area. One-way ANOVA, Tukey’s multiple comparison test, *n* = 10–12. ANOVA, analysis of variance; L, lumen; WTD, Western-type diet.

### Protease-Activated Receptor 2 Expression in Plaques Correlates with Oxidative Stress

PAR2, the receptor for coagulation proteases FXa and FVIIa, is known to be involved in the progression of atherosclerosis, and PAR2 deficiency has been shown to attenuate atherosclerosis in mice. 11 Therefore, aortic tissues were stained and quantified for PAR2.

Total PAR2 expression was significantly decreased in all anticoagulation groups compared with the WTD group ( Fig. 7A ). The same was observed for PAR2 expression in both the vasculature ( Fig. 7B ) and in plaques ( Fig. 7C ). A significant increase in PAR2 expression in the vasculature of the switching group was observed compared with the continuous rivaroxaban treatment ( Fig. 7B ). Interestingly the opposite was seen in plaques, where in the switching group PAR2 expression shows a decreasing trend in PAR2 expression compared with the continuous rivaroxaban treatment ( Fig. 7C,D ).

**Fig. 7:**
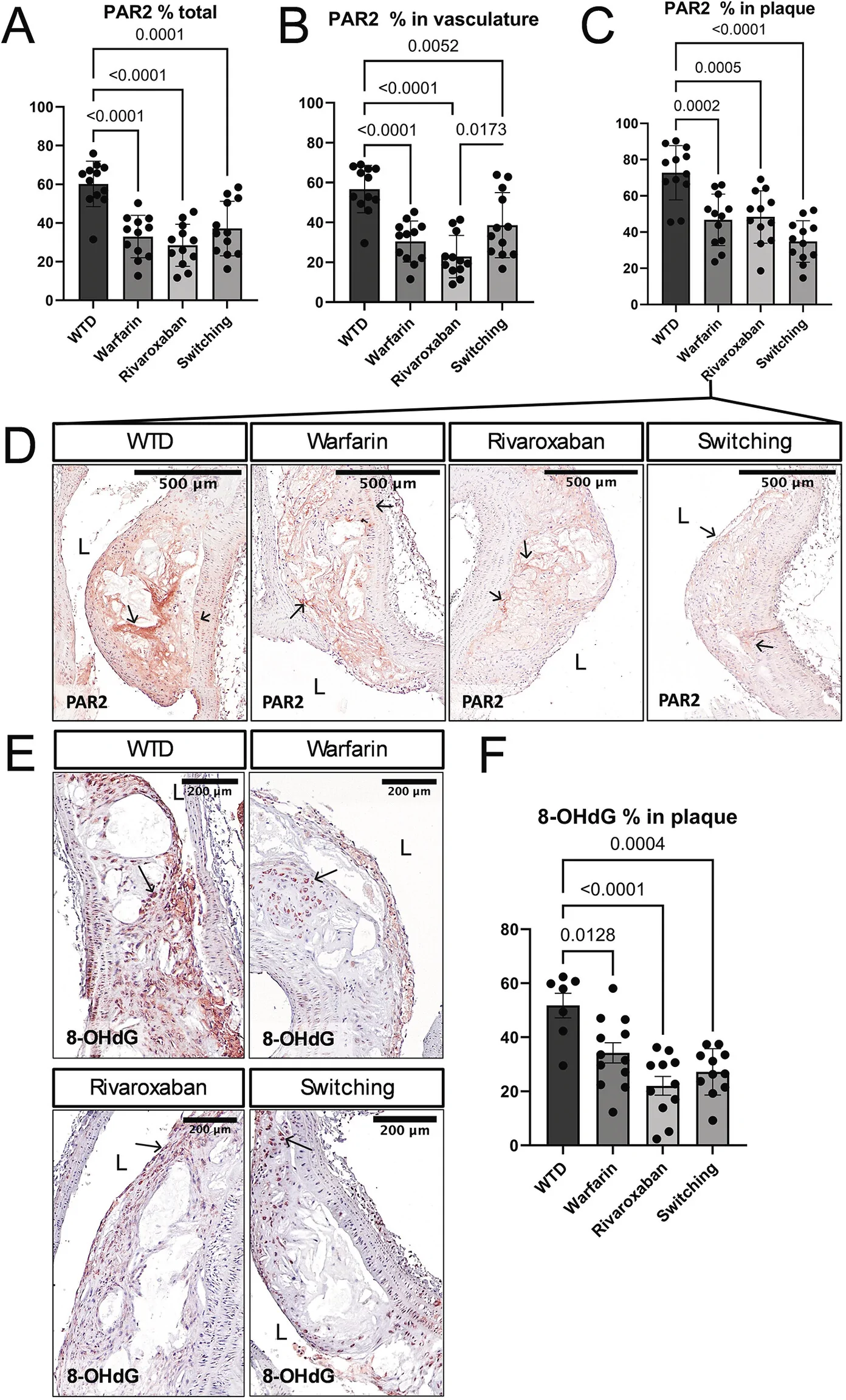
PAR2 expression and oxidative stress. ( **A** ) Quantification of PAR2 as a percentage of total signal over tissue area. One-way ANOVA, Tukey’s multiple comparison test, *n* = 12. ( **B** ) Percentage of PAR2 expressed only in the vasculature. One-way ANOVA, Tukey’s multiple comparison test, *n* = 12. ( **C** ) Percentage of PAR2 expressed in the plaques. One-way ANOVA, Tukey’s multiple comparison test, *n* = 12. ( **D** ) Representative pictures of plaques stained for PAR2. Scale bar = 500 μm. Black arrows indicate examples of positive signal. ( **E** ) Representative pictures of oxidative damage in atherosclerotic plaques stained using 8-OHdG. Scale bar = 200 μm. Black arrows indicate examples of positive signal. ( **F** ) Quantification of 8-OHdG signal as a percentage of plaque size. One-way ANOVA, Tukey’s multiple comparison test between the anticoagulation groups, *n* = 7–12. ANOVA, analysis of variance; L, lumen; PAR2, protease-activated receptor 2; WTD, Western-type diet; 8-OHdG, 8-Hydroxydeoxyguanosine.

Lastly, increased oxidative stress is a known hallmark of vulnerable plaques. 16 Aortic arches were stained for 8-Hydroxydeoxyguanosine (8-OHdG), a marker of oxidative DNA damage ( Fig. 7E ). Interestingly, expression levels of 8-OHdG in all groups were comparable with total PAR2 expression levels. In the anticoagulation groups, both PAR2 and oxidative stress were reduced ( Fig. 7F ) compared with the WTD group. Furthermore, the continuous rivaroxaban treatment visibly showed the lowest vascular oxidative stress compared with the other groups.

## Discussion

In this study, we investigated whether transitioning from warfarin to rivaroxaban could beneficially influence plaque composition and vessel wall remodelling. First, we validated the anticoagulation therapies. The thrombin generation curves obtained are consistent with the established pharmacodynamic differences between vitamin K antagonists and direct FXa inhibitors. Warfarin reduces the hepatic synthesis of the vitamin K-dependent clotting factors, particularly prothrombin, thereby limiting the total substrate available for thrombin generation. 17 Consequently, although peak thrombin levels may be reached, the propagation phase is shortened, resulting in a narrower thrombin generation curve and reduced ETP. In contrast, rivaroxaban directly inhibits FXa within the prothrombinase complex, primarily slowing the rate of thrombin formation rather than reducing substrate availability. 18 This kinetic modulation delays and flattens the thrombin burst, producing lower peak heights but a broader curve, which may preserve overall ETP at clinically relevant concentrations. Importantly, thrombin peak height influences fibrin clot architecture: high peaks (even at similar ETP) drive the formation of dense networks composed of thin fibrin fibers that are relatively resistant to fibrinolysis, whereas lower peaks favor thicker fibers and a more lysis-prone structure. 19 This suggests that rivaroxaban may promote a less thrombogenic clot phenotype beyond its effects on overall thrombin generation.

Second, in *Apoe ^−/−^* mice, continued rivaroxaban treatment reduced the progression of plaque size and calcification relative to warfarin, with the slowest progression observed in the switching group. We hypothesize that short-term warfarin exposure could be protective through its global suppression of coagulation, which may in turn reduce PAR2 activation and signaling. Consistent with this, PAR2 staining showed significantly higher expression in the non-anticoagulated group (WTD-only) compared with the anticoagulation groups, suggesting that reduced coagulation activation is associated with reduced PAR2 expression. Six weeks of warfarin treatment may confer a greater protective effect than rivaroxaban; however, this early protective effect appears to be offset by warfarin’s long-term negative effects on the vasculature.

Regarding vessel wall remodelling, rivaroxaban, particularly in mice first treated with warfarin, was associated with decreased arterial stiffness and improved structural integrity, evidenced by fewer elastic fiber disruptions. Moreover, switching from warfarin to rivaroxaban promoted features of plaque stabilization, including a higher abundance of αSMA-positive VSMCs and increased collagen deposition, both indicative of reduced rupture risk. Both rivaroxaban-treated groups exhibited lower intraplaque MMP-9 levels, underscoring its protective effects against plaque progression and stability. Finally, PAR2 expression and oxidative stress were reduced under all anticoagulation regimens, with the switching group showing the most pronounced decrease in PAR2, further supporting enhanced plaque stability.

Posthuma et al investigated the reversing effects of rivaroxaban on atherosclerotic plaque progression in *Apoe ^−/−^* mice. 10 This study involved 14 weeks of WTD feeding, followed by a 6-week treatment period with either WTD alone or WTD supplemented with rivaroxaban. Their results demonstrated that switching to rivaroxaban for 6 weeks after 14 weeks of WTD reduced overall plaque burden and enhanced features of plaque stability. In contrast, our experimental design implements rivaroxaban following a 6-week regimen of WTD combined with warfarin. Nevertheless, plaque size was similarly reduced in the rivaroxaban-switching group compared with the continuous warfarin group. Given that plaque size at 6 weeks in warfarin-treated animals was already significantly greater than in the WTD and rivaroxaban groups, it further supports that rivaroxaban may exert plaque-regressing effects. Additionally, we conducted analyses of vessel wall stiffness and elastin fiber integrity to evaluate the impacts of different anticoagulation therapies on the vasculature. The switching group exhibited the lowest vascular stiffness with fewer elastin fiber breaks, indicating a more favorable outcome on vascular integrity. These findings are in-line with previous work showing that warfarin was associated with an increased number of elastin breaks. 20 Notably, PWV was significantly reduced in the switching group only and not in the continuous rivaroxaban group. We hypothesize that this effect is driven by differences in VC rather than by the coagulation system itself. The switching group showed lower calcification than the continuous rivaroxaban group, and this difference in calcium deposition may account for the observed differences in PWV and elastin fragmentation. Calcification deposits located on or near elastic lamellae increase local mechanical stress and increase the susceptibility of elastin fibers to enzymatic cleavage. 21 Under this interpretation, the effect might reflect the calcification state of the vessel wall rather than a direct anticoagulant effect.

VSMCs undergo phenotype switching during plaque formation and vascular remodelling, losing contractile markers such as αSMA and SM22α, and acquiring markers associated with synthetic, osteogenic, or macrophage-like phenotypes, including osteopontin and CD68. 22 To date, multiple VSMC synthetic phenotypes have been identified and described in atherosclerotic plaques. 23 Given the heterogeneous nature of VSMC-like phenotypes in plaques, we measured αSMA expression to identify contractile VSMCs. αSMA-positive VSMCs in the fibrous cap surrounding the atherosclerotic plaque were identified in the switching group, a pattern associated with greater plaque stability 24 that was not observed in the other treatment groups. VSMCs in the atherosclerotic plaques are responsible for synthesizing collagen; the amount and the matrix organization of collagen influence the mechanical stability of the fibrous cap. 25 The presence of increased plaque surrounding VSMCs in the switching group is consistent with the slightly increased collagen content in the plaque. This convergence of increased contractile VSMCs and enhanced collagen deposition strongly indicates a shift toward a more stable, fibrous plaque phenotype selectively driven by the removal of warfarin’s detrimental vascular effects combined with subsequent FXa inhibition. These data further support the beneficial effect of switching from warfarin to rivaroxaban on plaque stability.

The expression of MMPs is a critical determinant of plaque stability, as proteolytic enzymes contribute to ECM degradation and structural weakening of the vessel wall. Specifically, MMP-9 has been identified as a predictor of increased atherosclerotic plaque instability. 26 Elevated MMP-9 expression is mainly found at the rupture-prone shoulder regions of human vulnerable atherosclerotic plaques. 27 Given the critical role of MMP-9 in determining plaque rupture risk, we performed immunostaining of aortic tissues to assess MMP-9 expression. Our findings indicate that both rivaroxaban groups showed substantially reduced MMP-9 expression within plaques. Activated coagulation factors, such as FVIIa and FXa, possess signaling properties by activating PAR2, expressed on the surface of vascular cells. 28 Moran et al demonstrated that inhibiting FXa with fondaparinux reduced atherosclerosis and suppressed PAR2 expression in the suprarenal aorta. 29 Consistent with this, PAR2 deficiency in an atherosclerosis-prone low-density lipoprotein receptor-deficient ( *Ldlr ^−/−^* ) mouse model attenuated plaque burden and diminished the release of inflammatory chemokines from VSMCs. 11 Together, these findings highlight that targeting coagulation factor signaling through PAR2, or blocking PAR2 itself, confers protective effects on atherosclerotic plaque development. Our results further support this concept. PAR2 expression was reduced in both the vasculature and plaques across all three anticoagulation regimens compared with the WTD group, with the most pronounced decrease observed in the plaques of the switching group. This aligns with the reduced VSMC phenotypic switching observed in this group, reflected by increased αSMA within plaques, since elevated PAR2 expression is typically associated with a more synthetic, destabilizing VSMC phenotype. 30

Oxidative stress is a major driver of plaque vulnerability, and multiple coagulation factors have been implicated in promoting oxidative injury. 16 Inhibition of FXa with edoxaban has been shown to reduce oxidative stress, while blockade of PAR2 effectively abolished FXa-induced reactive oxygen species (ROS) production in human proximal tubular cells. 31 Because FXa catalyzes the conversion of prothrombin to thrombin, and thrombin itself can directly induce oxidative stress in both human and rat VSMCs, 32 targeting upstream coagulation factors has the potential to interrupt these pro-oxidant pathways. Supporting this, dabigatran has been reported to mitigate thrombin-induced oxidative stress in cultured human VSMCs. 20 Consistent with these mechanistic insights, our study demonstrates that all three anticoagulation regimens significantly reduced oxidative damage compared with the WTD group, underscoring the role of coagulation-dependent signaling in driving oxidative stress. Notably, continuous rivaroxaban treatment showed the lowest degree of oxidative damage relative to WTD, suggesting that direct FXa inhibition, and the consequent reduction in thrombin inhibition, offers substantial protective benefit for maintaining plaque stability.

Taken together, our findings demonstrate that anticoagulation therapy consistently reduces both PAR2 expression and oxidative damage within atherosclerotic plaques. In contrast, the detrimental effects observed under warfarin treatment, including accelerated plaque growth, increased calcification, and elevated MMP-9 levels, underscore the superiority of rivaroxaban in promoting plaque stability. Notably, switching from warfarin to rivaroxaban yielded the most favorable outcomes, suggesting that the removal of warfarin-driven vascular stress combined with direct FXa inhibition confers additive protective effects. Although clinical validation is required, these results carry important implications for optimizing anticoagulant strategies, particularly in patient populations where plaque vulnerability and coagulation-driven inflammation critically shape cardiovascular risk.

## Declaration of GenAI Use

During the writing process of this paper, the authors used Claude AI to check grammar and syntax. The authors reviewed and edited the text and take full responsibility for the content of the paper.
